# Hepatitis E Virus Infection in Solid Organ Transplant Recipients, France

**DOI:** 10.3201/eid2302.161094

**Published:** 2017-02

**Authors:** Sebastien Lhomme, Laurent Bardiaux, Florence Abravanel, Pierre Gallian, Nassim Kamar, Jacques Izopet

**Affiliations:** Institut National de la Santé et de la Recherche Médicale, Toulouse, France (S. Lhomme, F. Abravanel, N. Kamar, J. Izopet);; Centre Hospitalier Universitaire de Purpan, Toulouse (S. Lhomme, F. Abravanel, J. Izopet);; Université Paul Sabatier, Toulouse (S. Lhomme, F. Abravanel, N. Kamar, J. Izopet);; Etablissement Français du Sang Pyrénées Méditerranée, Toulouse (L. Bardiaux);; Etablissement Français du Sang Alpes Méditerranée, Marseille, France (P. Gallian);; Ecole des Hautes Études en Santé Publique, Marseille (P. Gallian);; Centre Hospitalier Universitaire de Rangueil, Toulouse (N. Kamar)

**Keywords:** hepatitis E, blood transfusion, solid organ transplant patients, hepatitis E virus, viruses, France

## Abstract

The rate of transfusion-transmitted hepatitis E virus (HEV) in transplant recipients is unknown. We identified 60 HEV-positive solid organ transplant patients and retrospectively assessed their blood transfusions for HEV. Seven of 60 patients received transfusions; 3 received HEV-positive blood products. Transfusion is not the major route of infection in this population.

Hepatitis E virus (HEV; family *Hepeviridae,* genus *Orthohepevirus*) is a single-stranded, positive-sense RNA virus of ≈7.2 kb. At least 4 genotypes are responsible for hepatitis E in humans (HEV-1–4). HEV-1 and HEV-2 infect only humans, while HEV-3 and HEV-4 have animal reservoirs (*1*). In developed countries, the main source of HEV transmission is the consumption of raw or undercooked, infected meat or direct contact with infected animals. Cases of bloodborne transmission have also been reported (*1*–*3*). 

Transfusion-transmitted infections in solid organ transplant (SOT) patients remains a major concern; the frequency at which these infections occur is unknown (*4*). SOT patients receiving transfusions are at risk of contracting HEV because systematic screening for the virus is rare. For SOT patients exposed to HEV, infection can become chronic, with rapidly progressing liver disease (*1*). Because of the high incidence of HEV infection in the Midi-Pyrénées area (*1*), SOT patients are regularly screened for HEV RNA, and diagnosis of HEV infection is made at the time of alanine aminotransferase elevation.

We investigated retrospectively the extent to which transfusion-transmitted HEV infections occurred in a cohort of 60 SOT patients infected with HEV from January 1, 2009, through June 30, 2014. We found that 7 (11.7%) of these SOT patients were potentially infected through transfused blood products because they were given transfusions in the 6 months preceding the diagnosis (Technical Appendix Table 1); the remaining 53 HEV-positive patients were infected by other modes. The median HEV RNA concentration in recipient blood was 5.4 log copies/mL (range 3.6–6.8 log copies/mL) or 5.2 log IU/mL (range 3.4–6.6 log IU/mL). The median interval between transfusion and diagnosis was 4 months (range 0.2–5.0 months). HEV infections developed in 4 patients (R1, R3, R4, and R5) 6 months after transplantation. Transmission of HEV by the graft was excluded in these patients by examining the samples from the organ donors at the time of donation. None of them tested positive for HEV RNA. 

We collected the 231 blood samples corresponding to the 7 patients’ donors (stored by the French Blood Agency) and tested them individually for HEV RNA and HEV IgM/IgG (Technical Appendix). Of these samples, 7 (3.0%) tested positive for HEV RNA (Technical Appendix Table 2). This analysis revealed that 3 patients (recipients R1, R2, and R3) received ≥1 blood components derived from the 7 HEV RNA-positive donations; 4 patients were not given viremic donations. 

The median HEV RNA dose given to the recipients was 5.1 log copies (range 3.8–8.4 log copies) or 4.9 log IU (range 3.6–8.2 log IU). Recipient R1 received blood components from 1 viremic donor (D1), while R2 and R3 received blood components from 3 HEV RNA-positive donors (D2.1–D2.3 and D3.1–D3.3, respectively). Phylogenic analyses of the 348-nt partial sequences of the open reading frame (ORF) 2 region (Technical Appendix) showed that R1/D1 and R3/D3.1 sequences clustered together (Figure); nucleotides were >99.0% identical in both cases, confirming transfusion-transmitted HEV infection. Phylogenetic analysis of R2/D2.3 showed they clustered together but had a lower sequence identity (84.2%), suggesting transmission could have been mediated by another mechanism.

**Figure:**
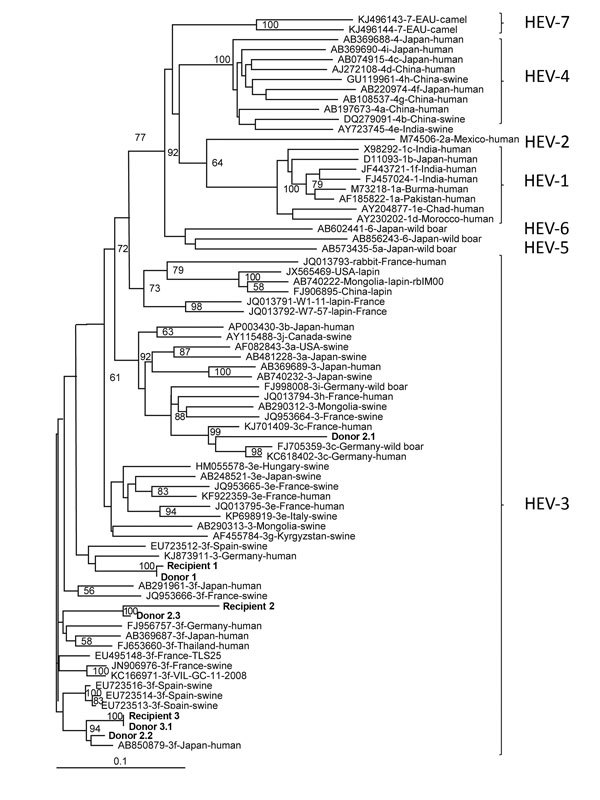
Phylogenetic tree of hepatitis E virus (HEV) isolates from 3 HEV-positive blood donors and 3 solid organ transplant recipients (shown in bold), France, compared with reference isolates. The tree was constructed by using partial open reading frame 2 sequences (348 nt). HEV genotypes are indicated at right. A confirmed case of transfusion-transmitted HEV infection requires evidence of infection in the recipient and donor and that the nucleotide sequences of these isolates be identical. The isolates from France were deposited in GenBank under accession nos. KX452928–KX452935; accession numbers, sources, and location of isolation for other isolates are indicated. Scale bar indicates nucleotide substitutions per site.

Another study conducted from January 2004 through June 2009 in France found that the risk factors associated with HEV transmission in SOT patients were eating pork meat, game, and mussels (*5*). Thus, the risk for transfusion-transmitted HEV infection is lower than the risk for acquiring an HEV infection from other sources in the environment in this population. Our study supports these results; we identified viremic donors as the source of infection in 2 (or possibly 3) of 60 HEV-positive SOT patients using phylogenetic analyses.

In France, HEV-positive samples were found in 1/2,218 blood donations, with HEV RNA concentrations of <60 to 29,796 IU/mL (*6*). In the Netherlands, 1/2,671 donations was viremic, with HEV RNA concentrations from <25 to 470,000 IU/mL (*7*). Published data indicate that the minimum infectious dose in donations is 7,056 IU (3.85 log IU) (*8*). A recent study found that donations associated with HEV-transmission had higher HEV RNA concentrations than did those that were not associated with HEV transmission (*9*). In our study, the 3 blood donations implicated in HEV transmission had HEV RNA doses >5.7 log copies (i.e., 5.5 log IU). Another parameter that must be considered is the presence of HEV antibodies in the donor or in the recipient, although the concentration needed to protect against an HEV infection is still unclear.

We conclude that, although transfusion-transmitted HEV infection can occur in SOT patients, blood transfusion is not the main source of transmission in these patients in France. Optimal policies for screening blood donations for HEV must be defined according to epidemiologic data.

Technical Appendix Methods used to characterize hepatitis E virus (HEV) infections and characteristics of the HEV-positive blood products obtained from donors and the solid organ transplant patients who received the contaminated blood components.
