# Supplementary material for: Hepatitis E Virus Infection in Solid Organ Transplant Recipients, France
Source: Emerg Infect Dis. 2017 Feb;23(2):353–6. doi: 10.3201/eid2302.161094 (PMC5324811; doi:10.3201/eid2302.161094)
Supplement: Technical Appendix — Methods used to characterize hepatitis E virus (HEV) infections and characteristics of the HEV-positive blood products obtained from donors and the solid organ transplant patients who received the contaminated blood components. [file 16-1094-Techapp-s1.pdf]

Article DOI: http://dx.doi.org/10.3201/eid2302.161094

Hepatitis E Virus Infection in Solid Organ Transplant Recipients, France

Technical Appendix

Methods

Detection of Hepatitis E Virus (HEV) Antibodies

Anti-HEV IgM/IgG were detected by enzyme-linked immunosorbent assay (Wantai, Beijing, China).

Plasma HEV RNA Concentrations

HEV RNA in donor blood was detected and quantified by using a validated real-time PCR protocol with a detection limit of 100 copies/mL (i.e., 60 IU/mL) (*1*).

HEV Genotype Determination

HEV was genotyped by sequencing a 348-nt fragment within the open reading frame (ORF) 2 gene (*2*). The sequences were compared with reference HEV sequences (*3*). A confirmed case of transfusion-transmitted HEV infection requires evidence of infection in the recipient and a component of the blood from the donor testing positive for HEV RNA. It also requires that the nucleotide sequences of the 2 isolates be identical.

Technical Appendix Table 1. Characteristics of the 7 transplant recipients potentially infected with HEV through blood transfusion, France

Recipient	Sex	Organ transplanted	Age at ALT elevation	HEV antibody titer before transfusion,* U/mL		Interval between event and ALT elevation, mo		HEV RNA concentration at ALT elevation, log copies/mL	Genotype
				IgG	IgM	Transfusion	Transplantation		
R1	F	Liver	34	–	–	4	3	5.9	3
R2	M	Kidney	41	–	–	1	42	3.6	3f
R3	F	Liver	42	–	–	4	4	6.6	3f
R4	F	Liver	57	+, 0.25	–	4	4	5.4	3f
R5	F	Kidney	65	–	–	4	4	4.1	3f
R6	M	Heart	44	–	–	5	7	6.8	3f
R7	F	Liver	55	+, 10.97	–	0.2	82	5.1	3c

ALT, alanine aminotransferase; HEV, hepatitis E virus.

Technical Appendix Table 2. Characteristics of the 7 blood donors with HEV RNA–positive, archived blood samples, France

Donor/ recipient	HEV RNA concentration, log copies/mL	Geno type	Anti-HEV IgG titer, U/mL	Anti-HEV IgM	HEV-positive blood component	Estimated transmitted volume, mL	Transfused dose of HEV RNA, log copies
D1/R1	4.7	3	+	+	Red blood cell unit	10	5.7
D2.1/R2	2.8	3c	–	–	Red blood cell unit	10	3.8
D2.2/R2	3.3	3f	+, 0.37	+	Pooled platelet concentrate	67	5.1
D2.3/R2	6.6	3f	not determined	not determined	Pooled platelet concentrate	63	8.4
D3.1/R3	4.3	3f	–	–	Amotosalen-treated plasma	209	6.6
D3.2/R3	3.0	Na	+, 2.97	+	Pooled platelet concentrate	69	4.8
D3.3/R3	2.6	Na	–	–	Pooled platelet concentrate	67	4.4

HEV, hepatitis E virus; Na, not available due to low HEV RNA concentration.

References

1. Abravanel F, Sandres-Saune K, Lhomme S, Dubois M, Mansuy JM, Izopet J. Genotype 3 diversity and quantification of hepatitis E virus RNA. J Clin Microbiol. 2012;50:897–902. PubMed http://dx.doi.org/10.1128/JCM.05942-11

2. Legrand-Abravanel F, Mansuy JM, Dubois M, Kamar N, Peron JM, Rostaing L, et al. Hepatitis E virus genotype 3 diversity, France. Emerg Infect Dis. 2009;15:110–4. PubMed http://dx.doi.org/10.3201/eid1501.080296

3. Smith DB, Simmonds P, Izopet J, Oliveira-Filho EF, Ulrich RG, Johne R, et al. Proposed reference sequences for hepatitis E virus subtypes. J Gen Virol. 2016;97:537–42. PubMed http://dx.doi.org/10.1099/jgv.0.000393
